# The Cyclic Value-Context Reinforcement Model of Problematic Internet Use: Empirical Validation Using a Thematic Analysis of Children’s Counseling Data

**DOI:** 10.2196/17996

**Published:** 2020-07-14

**Authors:** Young Yim Doh, Bugeun Kim, Seul Lee, Gahgene Gweon

**Affiliations:** 1 Graduate School of Culture Technology Korea Advanced Institute of Science and Technology Daejeon Republic of Korea; 2 Graduate School of Convergence Science and Technology Seoul National University Seoul Republic of Korea; 3 Advanced Institute of Convergence Technology Suwon Republic of Korea

**Keywords:** problematic internet use, children, cyclic value context reinforcement model, psychosocial value, environmental context, internet utility

## Abstract

**Background:**

Research on problematic internet use has focused on devising diagnostic criteria or describing the factors that influence internet overuse. However, a paradigm shift is necessary in studying the phenomenon of increased internet use not just from a pathological point of view but also from a developmental point of view that considers children’s behavior of adapting to a technology-oriented society.

**Objective:**

In this paper, we propose the Cyclic Value-Context Reinforcement Model (CVCRM) to understand problematic internet use behavior. The purpose of our study was to construct a developmental process model that provides a holistic understanding of problematic internet use behavior of children and to empirically validate the proposed model by conducting a thematic analysis on actual counseling data.

**Methods:**

To validate the CVCRM, we conducted thematic analysis using the counseling data from 312 Korean children aged 7-18 years. For the coding process, 7 master’s and doctoral student researchers participated as coders, and 2 professors supervised the coding process and results.

**Results:**

This project was funded from October 2015 to September 2019 to analyze counseling data from 312 children who participated in counseling sessions during January 2012 to May 2014. Based on the data analysis, we present the CVCRM, which integrates existing theoretical approaches and encompasses the 3 interacting aspects that induce and reinforce problematic internet use in children: psychosocial value, environmental context, and internet utility. Specifically, using counseling data, we empirically ascertained that problematic internet use behavior feeds into children’s psychosocial values and environmental contexts, which in turn facilitates problematic internet use in a cyclical manner.

**Conclusions:**

Through this empirical validation, the CVCRM can provide a theoretical framework and an integrated perspective on the developmental mechanism of problematic internet use behavior of children.

## Introduction

### Background

The internet is pervading every aspect of daily life, even for children. Based on the European Union Kids Online Model of Children, Livingston et al [1] argued that the research agenda on children’s internet usage needs to shift direction from “how children engage with the internet as a medium” to “how children engage with the world mediated by the internet.” Furthermore, they raised a critical research question: “For which children under which circumstances does internet use lead to risk and why?” This question redirects our attention from the internet itself to children as active agents, to the environmental context in which children are located, and to children’s developmental process of adaptation.

With the widespread use of the internet, problematic internet use is an important social issue in the global society. Compared to the term internet addiction or dependency, problematic internet use is broad enough to cover the negative consequences of the varying degrees experienced by an individual without the risk of social stigma as a pathological person [2-4]. Problematic internet use is defined as “use of internet that creates psychological, social, school, and/or work difficulties in a person's life” [5]. Until now, most research on problematic internet use has focused on devising diagnostic criteria or describing the factors that influence internet overuse. However, a paradigm shift in the study of the phenomenon of increased internet use is necessary, not just from a pathological point of view but also from a developmental point of view that considers children’s behavior of adapting to a technology-oriented society.

In this study, we propose the Cyclic Value-Context Reinforcement Model (CVCRM) to explain problematic internet use behavior. The purpose of our study was to construct a developmental process model that provides a holistic understanding of problematic internet use behavior of children and to empirically validate the proposed model by conducting a thematic analysis of counseling data collected from 312 Korean children, aged 7 to 18 years. We explored the adaptive functions of the internet-mediated environment in achieving children’s developmental tasks and satisfying their psychosocial values as well as their environmental needs.

### Problematic Internet Use Behavior: Need for an Integrated Process Approach

Research topics on problematic internet use behavior have changed from a predominantly symptom-centered approach to a cause-centered approach and from individual factors to environmental contexts that impact internet use. Using the symptom-centered approach, researchers developed diagnostic criteria based on the investigation of symptoms of internet overuse and designed intervention methods to relieve the corresponding symptoms [6-10].

However, a symptom-centered approach is not enough to change the causal context and prevent symptom development. To address this issue, a cause-centered approach has been established to identify factors affecting problematic internet use. Early cause-centered studies examined individual internal factors, including comorbidity [11-13] or psychological vulnerabilities, such as loneliness and shyness [14-16], as the cause of problematic internet use. In addition to the individual internal factors, research on external variables and environmental contexts, which include the influences of family, school, and society, has begun recently. Family function, communication, and family conflicts are important factors that affect problematic internet use [17-20]. Additionally, people with internet-related problems tend to experience more real-life problems in social relationships and school life [21,22]. Considering these results, in order to gain a more comprehensive understanding of the phenomenon, we need to examine environmental contexts as important factors in models that examine problematic internet usage.

Although existing work has identified a correlation between specific individual and environmental factors and internet use, we need to look beyond individual relationships between specific factors and consider the overall development of problematic internet use behavior. In particular, certain individual factors transact cyclically with other factors, usage behavior, and problematic symptoms. For example, depression, loneliness, and self-control have been found to interact with other factors that affect problematic internet use [14,23-25]. Thus, comorbidities and psychological vulnerabilities are causes, as well as consequences, of problematic internet use [26,27]. Kardefelt-Winther [27] also reported that the correlations of loneliness and social anxiety with excessive online gaming were no longer present when controlling for stress. In addition, symptoms of psychopathology mediate irrational beliefs and internet gaming addiction [28]. Therefore, the relationship between internet use behavior and factors influencing it can be transactional and cyclic, rather than a one-way causal relationship.

Given the expanded research interest, from symptoms to causes of problematic internet use behaviors and from individual factors to environmental contexts, a process-centered approach integrating these factors is necessary to increase an overall understanding of problematic internet use. In other words, from a holistic perspective, we need to understand the intersection between the environmental context and personal characteristics and to reveal the mechanism underlying how these factors transact with each other and reinforce problematic internet use behavior.

### Previous Theoretical Models on Problematic Internet Use Behavior

In order to construct an integrated developmental model and to guide our understanding in a qualitative thematic analysis, we selected 7 related theoretical models: (1) cognitive-behavioral model of pathological internet use [10], (2) a new model of media attendance [29], (3) model of compensatory internet use [30], (4) basic model of problematic internet use in youth [31], (5) differential-susceptibility to the media effects model [32], (6) reinforcing spirals model [33], and (7) player’s value structure in digital games [34]. In this section, we compare these 7 models and discuss the insights gained in relation to problematic internet use behavior.

Davis [10] introduced the cognitive-behavioral model of pathological internet use. This model described the following 3 categories of factors contributing to behavioral symptoms: individual psychopathology, internet exposure experience, and social deficits. The core factor of this model is “maladaptive cognitions.” Accordingly, the model suggests that individuals who have maladaptive cognitions have a negative view of themselves and use the internet to gain positive responses from others. Based on the cognitive-behavioral approach, this model was an early attempt to explain the etiology of pathological internet use. However, this model has limitations in that it focuses on the vulnerability of the individual by regarding internet use behavior as a pathological symptom. Another limitation of the model is that it considers the individual as a passive and socially deficient being that simply responds to situational cues.

LaRose and Eastin [29] proposed an internet use process model that combines the concepts of use and gratification with the social cognitive theory, by matching similar dimensions in both models. The following 6 expected internet outcomes were identified: novel, social, activity, monetary, self-reactive, and status. This model also added the factors of self-efficacy and self-regulation to broaden the understanding of use and gratification. The internet use process model explains internet use behavior using the expected outcomes of internet use, rather than focusing on problematic symptoms or individual vulnerabilities. In addition, the model regards users as active subjects that select media according to expected outcomes (ie, gratification). However, this approach does not consider the preceding factors that lead to the expected outcomes of internet use behavior.

Kardefelt‐Winther [30] presented a model of compensatory internet use and explained internet use behavior through motivation that stems from unmet real-life needs. This model suggests that people use the internet as a stress-coping strategy. Furthermore, the model considers the real-life context of internet usage by introducing the concept of “coping motivation,” which regards reality-based difficulties as an important factor for inducing internet use. However, this model can only explain internet use behavior as a coping strategy for people who experience problems in a real-life context. Additionally, because motivation is relatively temporary and limited to a specific situation, the model is not suitable for explaining persistent internet use behavior.

As a more comprehensive model, Tam and Walter [31] suggested a basic model of problematic internet use for youth that is based on the literature from the past 10 years. They argued that problematic internet use is conceptualized, not as a unitary mental health condition, but rather as complex pathways of underlying psychological, developmental, ecological, and intrafamilial factors. This model distinguishes between regular internet use, problematic or heavy internet use, and pathological internet use or gaming addiction. Additionally, it separates the predisposing and protective factors that affect each state. However, while this model describes each of the subfactors affecting problematic internet use in detail, it has a limitation that it does not account for the interrelationships between the different factors.

In the literature on the effects of media, Valkenburg and Peter [32] introduced the differential-susceptibility to the media effects model. The model proposed that media effects are conditional and depend on the following 3 types of differential-susceptibility variables: dispositional, developmental, and social. These 3 differential-susceptibility variables affect the choice of media use. Additionally, the model distinguished the following 3 media response states: cognitive, emotional, and excitative. These 3 response states mediate the relationship between media use and media effects. Finally, this model adopted the transactional proposition, which suggests that media use, media response states, and differential-susceptibility variables are influenced by media effects. This model also provided insights regarding the relationship between media and nonmedia variables. However, because it deals with a microlevel analysis focused on individual media users, it is difficult to apply to design intervention methods of behavioral change on the level of user activity or environmental affordance.

Based on a system-theory perspective [35] and the social identity theory [36], Slater [33] elaborated the reinforcing spirals model. It recognized that media use serves as both an outcome and a predictor in many social processes and that media use in the contemporary society is a principal means to maintaining personal and social identities. One premise of this model is that the process of media selection and the effect of exposure to selected media are dynamic and ongoing. Therefore, this model considers that users tend to select certain types of media contents according to their social context, social identity, and prior attitudes. In addition, certain media content exposures have a subsequent impact on the strength and accessibility of social group identification, attitudes, and behaviors. In turn, the influence of subsequent media use continues to reinforce those associated elements of social identity, attitude, and behavior over time. The virtue of this model is highlighting the ongoing dynamic social process of media use behavior. However, social identity is not static and could vary according to the function of the environmental contexts and values of users.

Recently, in the domain of game studies and consumer research, Lin et al [34] proposed the structure of attribute-consequence-value chains of digital game players. Using soft-laddering interviews and a theoretical framework of means-end chains, they revealed game attributes that players direct their attention to, including attributes such as the connection system, popularity, graphic design, and diverse game genres. Depending on these attributes, players experience certain consequences, such as improved interactivity, cultivated logic and reflex, acquisition of an authentic experience, enhanced pleasure of senses, and utilized imagination. This model also identified the values that game players pursue, such as fun and enjoyment of life, sense of accomplishment, warm relationships, and excitement. This study provided us with insights in terms of the sequential process of users’ values, media attributes, and experiential consequences.

### Insights for Developing the Integrated Model of Problematic Internet Use Behavior

We gained two main insights from reviewing existing models on internet use behavior. First, early studies attempted to explain the preconditions that could lead to internet use behavior, mainly in terms of an individual’s psychosocial characteristics. However, recent studies have expanded the understanding of the interactions to include personal characteristics and environmental contexts. Therefore, when constructing a new model, it is necessary to cover all 3 basic aspects of internet usage behavior: the individual’s psychosocial values and environmental contexts as well as the interaction between these two components.

Second, early studies looked at internet use behavior as a temporary problem and attempted to identify the cause or symptom of the problem by considering a simple causal relationship. However, recent studies have proposed a cyclic nature to the relationship between variables, one which creates persistent reinforcement and leads to the repetitive use of the internet. Therefore, it is necessary to examine the process that explains the types of internet utility that lead to stable and continuous problematic internet use. In addition, the model should link internet use back to the factors that exist within the context of daily life.

Based on these insights, this study proposes a hypothetical model that integrates existing theoretical approaches related to problematic internet use behavior from a developmental perspective, termed the CVCRM. The first research question of this study was: “Can we construct an integrated process model to understand problematic internet use behavior holistically?” The second research question is: “Can we identify factors and processes that interact with problematic internet use behavior in the CVCRM using children’s counseling data?”

## Methods

### Counseling Data Collection

We conducted a thematic analysis using the counseling data provided by the National Information Society Agency in the Republic of Korea. The data comprised reports on counseling sessions from January 2012 to May 2014 with children who experienced internet-related problems. Each case consists of one initial interview and at least one follow-up counseling session, ranging from 1 to 5 sessions. During the first interview, counselors measured the severity of internet-related problems and examined the basic environmental and individual conditions of the client. The questions in the initial interview pertained to the client’s home and school environments, time of the first internet use and motivation, feelings before and after internet use, how family and school respond to internet use, consequences of internet use, and the level of internet addiction. The optional follow-up counseling sessions after the initial interview were not strictly structured, and the counselors identified the clients’ core problems and attempted interventions. During the entire counseling process, the counselors recorded the client’s comments, as well as details about the client, his or her family members, and his or her family environment. All counseling records were archived as digital documents. We reviewed 312 cases that met the following 2 conditions: (1) case data included an initial interview report and had ≥1 follow-up session records and (2) clients aged between 7 and 18 years. This range is defined as the Korean school age and comprises entry into elementary school to graduation from high school.

### Thematic Analysis

Thematic analysis [37] is a qualitative research method that enables us to discover and analyze patterns or themes in data. This methodology consists of 2 approaches: top-down theoretical approach and bottom-up inductive approach. The purpose of our study was to construct a new model by integrating existing theories and to verify the model using empirical data. Therefore, we performed a thematic analysis that incorporated both theoretical and inductive approaches. Using both top-down and bottom-up approaches iteratively allows researchers to reflect on both theory and data from various perspectives. As a result, one can gain a richer understanding when using such an iterative approach compared to using just a top-down or bottom-up approach. The detailed analysis process was as follows. First, we formulated the initial model based on the integration of previous theoretical approaches. Second, we verified our model through a thematic analysis with iteration between our model and empirical data. We used counseling data from children for this bottom-up process. Our research team read 312 cases of counseling records to identify the overall problem situation (Step 1: familiarization with the data). Then, we repeatedly read all the counseling records. Each sentence in a record was read to identify meaningful statements that indicate children’s psychosocial values, environmental contexts, internet utility, and internet use behavior (Step 2: generation of the initial code). We repeated this process 9 times until the identified themes were saturated (Step 3: searching for and reviewing themes). Finally, all researchers reached a consensus on defining themes (Step 4: defining and naming themes). We performed a thematic analysis at the latent level to understand and theorize the underlying structures and processes of problematic internet use behavior. To establish credibility in the analysis, we used the researcher triangulation approach [38]. For each of the coding processes, 3-7 master’s and doctoral student researchers participated as coders, and 2 professors supervised the coding process and results. The Gwet’s AC1 interrater reliability coefficient was calculated in the ninth iteration for each of the following main themes: Children’s Psychosocial Value (10 factors, mean 0.887), Environmental Context (13 factors, mean 0.827), and Internet Utility (6 factors, mean 0.912). In case of disagreement that arose after the coding process, all the coders discussed the case to reach a final agreement.

## Results

### CVCRM

We formulated the CVCRM of problematic internet use behavior based on the insights drawn from previous theoretical approaches. First, we tried to cover 3 basic aspects in our model: children’s psychosocial values, environmental contexts, and internet utility. Second, we incorporated repeated behavioral tendencies between internet utility and internet use in our model. Third, our model addressed the cyclic reinforcement process linkage between problematic internet use behavior and the person-environment interaction unit, which are deeply interconnected within children’s daily life.

**Figure 1:**
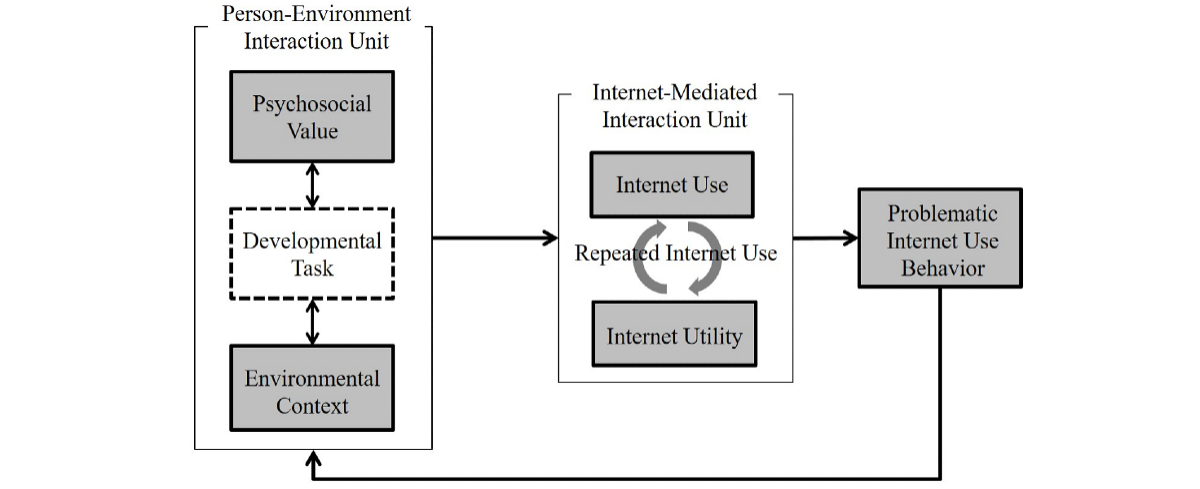
The Cyclic Value-Context Reinforcement Model of problematic internet use behavior.

Figure 1 shows the CVCRM. The “Person-Environment Interaction Unit” is an organism in which the children’s psychosocial values, developmental tasks, and environmental contexts interact. The specific conditions formed by the interaction of these elements can increase the probability of internet use. Internet use and internet utility are grouped into the “Internet-Mediated Interaction Unit.” They circulate and form a repeated internet use state. Repeated internet use leads to specific problematic internet use behavior, which can give feedback to the “Person-Environment Interaction Unit” to change its state. Through this process, internet use is induced and reinforced. The states of “Person-Environment Interaction Unit” and “Internet-Mediated Interaction Unit” are not static and may vary from person to person and from time to time. We defined and described each part comprising the CVCRM as follows.

#### Psychosocial Value

Psychosocial value is the intrinsic value of the child that is being met through internet use. As a result of our data analysis, 3 subthemes of psychosocial value appeared: individual, relational, and social. Psychosocial value represents relatively stable personal preferences as compared to “motivation or gratification” used in previous models. The themes and categories of the thematic analysis results are summarized in Table 1.

**Table 1:** Psychosocial value categories of problematic internet use behavior.

Subtheme and category		Category description	Example quotes
**Individual**			
	To relieve stressful events	Use the internet to alleviate or eliminate psychological stress such as tension, depression, loneliness, or anxiety.	“[Using the internet] relieves my stress.” “[When using the internet,] I’m not lonely anymore.”
	To express oneself	Evaluate the value of the internet as a means of expressing what one likes, one’s emotions, and one’s ideal self-image.	“[On the internet] I can express my opinions easily.”
	To experience a sense of accomplishment	Attach the value of the internet as a means of experiencing fulfillment, reward, and achievement.	“I feel a sense of accomplishment when I win.” “I enjoy raising my ranking in a game.”
	To spend one’s surplus time	Do not have any alternative activities other than surfing the internet and, thus, consider the internet as a convenient way to spend time.	“I get bored [when I come home], but there’s nothing else I can do [to spend my time].” “I play internet games when my parents don’t mind my time.”
	To express disobedience	Evaluate the value of the internet as a means of ignoring or actively resisting an authority’s instructions.	“I played internet games more since their [parents’] actions hurt my pride.” “I used the internet more heavily since my parents’ regulation on internet use caused more stress.”
	To escape from reality	Use the internet as a place to escape from real problems that cannot be fundamentally solved.	“I feel a sense of freedom since I can escape from school.” “Using the internet makes me forget about the severe conflict between my parents.”
**Relational/social**			
	To develop a sense of closeness	Evaluate the value of the internet as a means of interacting with particular subjects and maintaining familiarity.	“Internet enables me to keep in touch with my close friends who moved to other cities.” “My friend and I speak the same language because of the games that both of us play.”
	To participate in common activities in the peer group	Evaluate the value of the internet as a way to enter peer groups, follow the current trends, and collaborate with peers.	“I need the internet to hang out with my friends.” “It [the internet] makes me understand the world that my friends are in.”
	To be recognized as the best	Evaluate the value of the internet as a way to obtain authority, respect, and envy within a group.	“I’m proud when my friends admire me for my gaming skills.” “[Using the internet] makes me happy since many people online recognize me when I upload a drawing of a game character.”
	To experience controllability	Evaluate the value of the internet as a place where one can control one’s self, one’s group, and society.	“I want to relax, have some fun, … and keep everything under control.”

#### Developmental Task

Developmental task refers to what children must achieve at each stage of development. Thus, these tasks are fundamental psychosocial issues that can affect individuals of a certain age. Further, the developmental task at a specific age can affect and change one’s environmental contexts or psychosocial values. In the model presented in Figure 1, “the developmental task” is marked with a dotted box to indicate it is a latent variable. Life’s major roles are deeply interconnected with developmental tasks [39], as they refer to challenges or expectations that a culture has for individuals in different life phases [40]. The cyberspace, which is accessible via the internet, also serves as a channel for users to practice and achieve developmental tasks. For example, developing productive peer relationships is one of the important developmental tasks of school-aged children and teenagers, and it is more likely that they will have high value for social affiliation through internet use. However, the process in which these tasks interact with environmental contexts or individual value systems is not yet exposed. Therefore, the developmental task is regarded as a latent variable in this model.

#### Environmental Context

Environmental context includes the surrounding situation or environmental conditions that can affect the child’s perception or behavior. In the counseling data, 3 subthemes of environmental context appeared: individual, family, and society. Some environmental contexts can change with time and can be influenced by the outcome of internet use. However, each of the children’s experiences or perceptions of the environment, whether changed or not, may have an impact on his or her problematic internet use behavior. The themes and categories of the thematic analysis results are summarized in Table 2.

**Table 2:** Environmental context categories of problematic internet use behavior.

Subtheme and category		Category description	Example quotes
**Individual**			
	Situations of physical illnesses that limit daily life	Daily life is restricted by physical disability or illness.	“After being diagnosed with tuberculosis, I began to visit the PC cafe frequently while recuperating at home.”
	Situations of psychological vulnerability	Mental illness requiring professional treatment (eg, ADHD^a^, panic disorder) or psychological instability (eg, irritability, lethargy) is observed.	“[Mother says] client has social phobia.” “I’m depressed.” “I’m really worried about my school grades.”
	Situations of insufficient satisfaction with psychosocial needs	Complaints due to unsatisfied needs (eg, self-esteem), social exchanges (eg, social belongingness), or goal achievements (eg, realization of the ideal self) are observed.	“He doesn’t know how he can relieve his disappointment when his wishes cannot be granted.” “She [client] said she received art lessons but was forced to quit. She wants to pursue her career in art again.”
	Situations of lacking lifelong goals	A lack short-term or long-term goals or life commitments is observed.	“I don’t know what I should do [for the rest of my life].”
**Family**			
	Inadequate parenting	Caregiver’s undesirable parenting style, practice, or perspective, such as excessive regulation, insufficient caregiving, or physical or verbal violence, is observed.	“[Began using the internet excessively] after my mom didn’t allow me go outside.” “My parents don’t care [about my internet usage].” “Her [client’s] mother doesn’t have any faith in her.”
	Family communication problems	Weak family bonding or communication problems between family members are observed.	“My parents don’t get along. They fight over nothing.” “My parents only ask me to study. I want them to hear my thoughts about what I want to do.”
	Instability of the caregiver	Caregiver cannot nurture the client due to physical separation (eg, long business trips, divorce, or death).	“He [client] has a father who only visits on weekends.” “He [client] lives with his father after his parents got divorced when he was 6.”
	Internet use of other family members	Family members show problematic internet use.	“My mother is addicted to the internet.” “I cannot use the internet when my sister is at home since she is addicted to the internet.”
**Society**			
	Socially imposed stigma	Peer groups, schools, and the society insulate and isolate the client (eg, bullying, internet addiction, obesity, problem behavior).	“During middle school, he [a client] severely suffered from bullying.” “My family treats me as a mental patient.”
	Social or school maladjustment	Difficulty in social relations or social life or problematic behavior is observed.	“I’m afraid of meeting friends at school.” “He [a client] used the internet a lot because of social phobia and obsession problems.”
	Economic constraints	Client’s activity is limited due to economic constraints (includes client’s perception of economic constraints).	“The client said that he felt pity about himself, and described himself as being a beggar, several times throughout the week.” “I live in poverty.”
	Coexisting delinquent behavior	Delinquent acts (eg, runaway, truancy, drinking, smoking, sexual activity) or crimes (eg, school violence, assault, theft, robbery, or rape) are observed.	“The client bullied other students and often questioned the purpose of studying.” “His [client’s] teacher said she often had to search for him because he went to the PC café instead of attending school.”
	Unstructured daily activities	Client’s daily life is unstructured (no daily structure at home after expulsion or no regular activity plan for after-school hours).	“Since quitting the job at a restaurant, I spent time playing computer games [without any future plans].”

^a^ADHD: attention deficit hyperactivity disorder.

#### Internet Use

Internet use, or more specifically “repeated internet use,” refers to the state of repeated engagement in internet use activity while obtaining internet utilities.

#### Internet Utility

Internet utility satisfies the psychosocial value of a child, including the utility of content or relationships accessible via the internet. Internet utility can mediate the influence of the “Person-Environment Interaction Unit” on problematic internet use behavior to induce repeated internet use. In the counseling data, 3 subthemes of internet utility appeared: individual, relational, and social. The themes and categories of the thematic analysis results are summarized in Table 3.

**Table 3:** Internet utility categories of problematic internet use behavior.

Subtheme and category		Category description	Example quotes
**Individual**			
	To enjoy	Derive pleasure and fun from using the internet.	“Using the internet is the only pleasure in my life.” “The internet is thrilling.”
	To engage in goal-oriented learning	Establish goals to master specific skills or subjects in real life in the short or long term and gain learning effects over the internet.	“[When I’m using the internet,] I can code programs, which is both a hobby and a way of preparing for my future career.” “[On the internet,] there’re a lot of things that I can learn from.”
	To surf the internet	Browse and collect fragmentary information through the internet.	“It [the internet] helps with my homework.” “I used the internet to watch a show that a singer [who the client is a fan of] starred in, listen to his songs, and communicate with other fans.”
	To earn money	Acquire things that can make money or exchange money through the internet.	“I can earn a lot of money [through online gambling].” “I do online gambling because my allowances are not enough.”
	To satisfy one’s sexual curiosity	Satisfy one’s sexual curiosity through the internet.	“I sometimes watch pornography online.”
**Relational/social**			
	To be active in an online community	Communicate with new people through an online community or social networking service (including positive, neutral, and negative communication).	“It is fun to communicate with others anonymously.” “Arguing with others online is fun.”

#### Cyclic Reinforcing Process

Children’s psychosocial values, environmental contexts, and internet utilities interact to induce and reinforce internet use. Afterwards, problematic internet use is fed back into individual value systems and environmental contexts, resulting in repeated and strengthened internet use. We illustrate 5 representative counseling cases in high-risk groups that vividly reveal the cyclic reinforcing process in Table 4.

**Table 4:** Representative counseling cases that show the cyclic reinforcing process.

Case ID	Counseling content	Corresponding cyclic pattern
Case 57	“…I experienced school violence in middle school and became obsessed with games. […] I feel like I'm out of reality and feel liberated. I also feel like I am being recognized by people. […] The interpersonal relationship was so comfortable that I don’t feel the need to make friends offline. More recognition on the internet makes it more difficult to establish relationships in the real world…”	School violence (EC^a^) > escape from reality, social recognition (PV^b^) > enjoy relationship (IU^c^) > repeated and obsessive use (PIUB^d^) > weakening of offline relationships (EC) > cycle repetition
Case 155	“…Since I'm in the third year of high school, I have to study a lot, but I keep thinking about games. Eventually, I play games in the PC room with the excuse that I am going to the reading room on weekends. […] I'm not bored if I'm playing a game. I am anxious if I don't play games. I have to study, but I'm depressed and worried because I play a lot of games. […] I'm worried that, like my brother, I'm going to have a serious conflict with my parents…”	College entrance exam stress (EC) > relieve stress (PV) > enjoy fun (IU) > repeated and obsessive use, feeling of depression and worry (PIUB) > expectation of conflict with parents (EC) > cycle repetition
Case 295	“…Bored at home, I started the internet. […] I can use the internet to relieve stress and to kill time. I can meet new people in virtual space. In particular, I communicate well with people who play the same games. […] I tried to quit the game, but I was forced to play it again because of my clan friends…”	Unstructured daily life (EC) > relieve stress and spend surplus time (PV) > to be active in online community (IU) > excessive use (PIUB) > cycle repetition
Case 324	“…When I was in the third year of elementary school, my mom took me to a PC room. The internet is fun, stress-free, and pleasant. After that, I repeatedly went to the PC room to play games. […] I often went to PC rooms, and my mom's interference with games increased. Grandpa and grandma’s nagging got worse. I go back to the PC room to avoid it…”	Internet use of mother (EC) > relieve stress (PV) > enjoy fun (IU) > repeated visits to PC room (PIUB) > conflict with family members (EC) > cycle repetition
Case 559	“…Life is boring. When I'm immersed in the internet, I can be happy and have fun in my life. […] I spend a lot of time playing games. […] I went to the Rescue Internet Camp last year. At first it seemed to get better, but after a while it was back…”	Lack of a lifelong goal and insufficient satisfaction of psychosocial needs (EC) > to express oneself (PV) > enjoy and feel happy (IU) > repeated use (PIUB) > intervention in Rescue Internet Camp is useless (EC) > cycle repetition

^a^EC: environmental context.

^b^PV: psychosocial value.

^c^IU: internet utility.

^d^PIUB: problematic internet use behavior.

### Funding

This project was funded from October 2015 to September 2019 to analyze counseling data from 312 children who participated in counseling sessions during January 2012 to May 2014.

## Discussion

### Implications

Our proposal of the CVCRM of internet use behavior and our thematic analysis of the model using children’s counseling data have several implications. First, the CVCRM expanded the understanding of problematic internet use behavior by introducing the concepts of children’s psychosocial values and environmental contexts. In addition, the developmental task was included as a latent variable to explain the mechanisms of the developmental process of internet use behavior. In accordance with Lin et al [34], we adopted the concept of value based on the social adaptation theory by Kahle [41] and the suggested list of values [42]. Kahle listed 9 values (ie, self-respect, security, warm relationships with others, sense of accomplishment, self-fulfillment, sense of belonging, sense of being well-respected, fun and enjoyment of life, and excitement) and regarded these values as closely related to life’s major roles [42]. In addition, positive adaptation in children can be defined by achieving age-salient developmental tasks, and these tasks reflect the expectations and standards that parents, teachers, and society set for them [43]. Therefore, we need to consider children’s psychosocial values, developmental tasks, and environmental contexts in order to understand children’s problematic internet use behavior. In the counseling data, we found 3 subdimensions in the environmental context (ie, individual, family, and society). The structure of these 3 hidden dimensions is similar to the bioecological model of human development [44]. Bronfenbrenner and Morris [44] explained that both risks and resources for positive adaptation and development stem from factors situated within individuals (genetic and hormonal systems, personality, and cognition) as well as in the proximal (family and school) and distal (societal, cultural, and institutional) contexts in which their life is embedded [43].

Second, we proposed an extended understanding that internet use behavior is not a short-term problem state, but rather a persistently evolving and cyclically reinforcing process. At the surface level, the purpose of repeated internet use is obtaining internet utilities in the short term. However, if we widen our viewpoint to include the person-environment interaction unit, we can understand that internet utility originated from the interaction between children’s psychosocial values, developmental tasks, and environmental context needs. These multiple dimensions are relatively long-lasting characteristics, and the dimensions cyclically reinforce each other. Therefore, our study can contribute to the exploration of the cyclical nature of the relationship between variables that produce persistent reinforcement mechanisms of the repetitive use or re-use of the internet.

Third, the CVCRM can be applied to inform preventive interventions for problematic internet use. The current interventions tend to focus on directly changing personal characteristics or symptoms. However, personal characteristics, such as personality, self-regulation, self-efficacy, and cognition, are difficult to intervene and affect. Conversely, it is relatively easy to induce behavioral change by modifying environmental contexts. For example, in one case, a girl was constantly home alone because her parents both worked full-time jobs. She felt sad when her mother told her to “Take care of the problem yourself,” in response to trying to talk to her about a problem she faced. She used the internet for entertainment when she was home alone. Her internet usage duration increased, and she did not consider reducing it. Within a symptom-centered framework, she is at high risk of developing internet addiction. Here, therapists will attempt to intervene using a cognitive-behavioral approach, in which they will improve her self-regulation and plan her internet use time. However, from the value-context perspective, it is possible to change her parents’ attitudes to meet her needs of love and belongingness or to provide her with a structured daily life by offering opportunities to engage in cultural or leisure activities. Although the individual’s internal factors, such as self-control or depression, are abstract and difficult to change with short-term intervention, environmental contexts and value-seeking activities could change through practical interventions in a relatively short period.

### Limitations

There are some limitations in this study. The first pertains to the structure of the counseling data. Specifically, several factors may not have been discovered in the initial interview since the questions were semistructured. Furthermore, home-visit counseling sessions were loosely structured, so that counselors could freely talk and explore the client’s situation. The counseling record was not an exact transcript of the counseling session and was recorded as a summary based on the counselors’ comprehensive observations. Therefore, the counselors’ personal opinion may have influenced the records. Second, it is difficult to investigate cognitive factors because of the characteristics of children. Children’s self-consciousness is not yet fully developed. Without verbal statements, observations alone are not enough to identify abstract cognitive factors. Therefore, we did not include cognitive factors in the proposed model. However, cognitive factors, such as maladaptive cognitions [45] and desire thinking [46], are considered important predictors of internet use problems and may mediate the effects of values and contexts. Future research needs to explore the relationship between the factors proposed by the CVCRM and cognitive factors.

### Future Research

For future studies, we propose several directions to advance research in this domain. First, it is important to validate the present model using a quantitative approach given that our approach was an empirical validation. Furthermore, it is necessary to explore the interaction between environmental contexts and psychosocial values, as proposed by this study. Therefore, while we integrated these two concepts into the concept of the “person-environment interaction unit,” the detailed interactive processes taking place inside the unit are yet to be investigated. Second, there is a need to compare the changes in how the environment is perceived, psychosocial values, and internet utilities, based on developmental periods. By expanding the present study to the entire lifecycle, which includes young adults, middle-aged adults, and the elderly, we can explore the interaction between differential developmental tasks, environmental contexts, and value systems throughout the lifespan. Third, research is needed to compare the structures of the environmental contexts and psychological values surrounding internet use across various cultures. Values are not culturally neutral. For example, community connectedness is a strong value in the Korean society. Thus, social recognition and acceptance are important values in Korea for children to master, compared to other cultures where the value of independence may be encouraged as a virtue of adolescence. Lastly, developing specific counseling techniques and how such techniques can reinforce problematic internet use behavior are important and interesting topics to pursue. An indepth development of counseling strategies based on the CVCRM and the effects of such strategies would be a great direction for future work.

### Conclusions

We proposed the CVCRM of problematic internet use behavior from the developmental perspective using children’s counseling data. By performing a thematic analysis at the latent level, we could extend the current understanding of the underlying structure of internet use behavior. The core argument of our model is that children’s psychosocial values, environmental contexts, and internet utility interact to induce and reinforce problematic internet use. As a result, problematic internet use is reinforced back into the individual value system and environmental contexts, resulting in further strengthened internet use. Since the developmental task during adolescence can affect and change one’s environmental contexts or psychosocial values, it was included as a latent variable.

Human behavior is shaped by various factors, including those that underlie the environment. Once we understand the mechanisms by which children start and continue to use the internet, we can gain a deeper understanding of internet use behavior. Rather than attempting to change children’s internal traits or symptoms, it would be clinically useful to decipher and obtain the values that children seek and to create an environment that encourages them to change their behaviors on their own. From the perspective of developmental psychology, the value that children acquire by using the internet is related to a developmental task and is therefore very important in their growth process [40]. To help children derive value from the internet without falling into problematic use, a value-context approach may be useful in helping to create an environment in which children can recognize their own values regarding the internet and yet ultimately control and regulate their internet use behavior.

## Abbreviations

CVCRM: Cyclic Value-Context Reinforcement Model
EC: environmental context
IU: internet utility
PIUB: problematic internet use behavior
PV: psychosocial value
